# Environmental Radioactivity Monitoring and Measurements: Radon and Thoron

**DOI:** 10.3390/ijerph19159276

**Published:** 2022-07-29

**Authors:** Miroslaw Janik

**Affiliations:** National Institutes for Quantum Science and Technology, National Institute of Radiological Sciences, Inage, Chiba 263-8555, Japan; janik.miroslaw@qst.go.jp

We “bathe” in radiation, which is an integral part of our environment. All living beings are exposed to a flux of natural radiation, which is the primary source of human non-medical exposure to ionizing radiation. The most variable component of the population dose is exposure to naturally occurring radon and thoron gas as well as their progenies, which account for more than 50% of the total effective dose received from all sources of natural radiation (2.4 mSv annual dose) [1]. Recent epidemiological studies suggest that exposure to radon leads to an increased risk of lung cancer. An integrated strategy or action plan for national radon control is needed to reduce human exposure to radon.

In general, an action plan can be defined as a government document that defines strategy, principles, timetable, and regular evaluation. A National Radon Action Plan (RAP) should consist of five stages according to the flow chart for developing a RAP provided by the IAEA [2] They are as follows: (1) scientific interest in radon; (2) 1st phase—first assessment of the radon risk in the country; (3) 2nd phase—preparatory phase for the RAP; (4) 3rd phase—RAP implementation; and (5) 4th phase—RAP realization and evaluation. The ultimate goal of the RAP is to identify strategies to enact the necessary changes to reduce radon exposure. Strategies include requiring radon testing and reduction systems as standard practice in housing finance and insurance programs and institutionalizing radon risk reduction through building code requirements [3].

Currently, many countries are at Stage 1 and Stage 2 of the RAP. These stages require measuring concentrations of radon, thoron and progenies, as well as related environmental parameters and human behaviors. There are various available techniques, methods and instruments with advantages and disadvantages; therefore, their selection depends on needs and applications. Assessing radon levels allows for the optimal selection of mitigation techniques, if necessary, to reduce radon concentrations in residential and public spaces, from both legal and technical viewpoints.

The purposes of this Special Issue of “Monitoring and Measurement of Radioactivity in the Environment: Radon” are to present the results of efforts that have been made to monitor and measure radon as accurately and effectively as possible and to consider its impact on public health.

Therefore, reviews and original papers that explain current research interests and studies in radon and thoron have been collected. In general, the following two main topics were described: (1) metrology and calibration; and (2) measurement and dose assessment.

One of the most critical factors in radon measurement is the establishment of precise and accurate equipment calibration, which should be carried out to the highest possible internationally recognized standards. The European Radon Metrology and Monitoring Project (MetroRADON) included an inter-laboratory comparison to assess the metrological traceability of European radon calibration equipment and demonstrate its performance and precision in calibrating measuring instruments for radon in the range of 300 to 10,000 Bq·m${}^{-3}$. Beck et al. presented the results of an international intercomparison in which fifteen calibration facilities from twelve different European Union countries and one facility from Montenegro participated. Within the specified study range between 300 and 10,000 Bq·m${}^{-3}$, three different exposure levels with low (400 Bq·m${}^{-3}$), medium (1000 Bq·m${}^{-3}$) and high (6000 Bq·m${}^{-3}$) radon activity concentrations were defined for the comparison. The test quantity that was specified for a comparison between the facilities’ results was the ratio of the radon activity concentration, which was provided by each facility as an average value for a given exposure period, to the average radon activity concentration, which was measured during the same period with a comparison device. From this intercomparison study, it was concluded that the radon activity concentrations, determined by European calibration facilities that complied with metrological traceability requirements, were consistent with each other [4].

Within the MetroRADON project framework, the SUJCHBO laboratory in the Czech Republic performed and coordinated the intercomparison of secondary standards in the range of 200–300 Bq·m${}^{-3}$ [5]. Eight laboratories from eight European countries participated in this exercise. The z-score and the estimate of deviation (measurement error) were used for the quantitative comparison of results. Based on the evaluation of the parameters of each of the participating laboratories’ performance, it was judged that the secondary standards of the European calibration laboratories were met at an accurate level.

Typically, continuous monitors are used for radon and thoron calibration experiments, as well as to evaluate radon variability over short time intervals, such as 1 h. However, experimental results revealed that some active devices are susceptible to thoron, which can lead to unreliable and overestimated results. To test this phenomenon, Di Carlo et al. [6] evaluated the interference introduced by thoron presence on the radon concentration returned by four active devices exposed in the same atmosphere where radon and thoron coexisted at levels of the same order of magnitude. They found that some devices could be affected by thoron. However, they were able to propose an indirect method for estimating thoron concentration using devices that are not explicitly designed to measure thoron concentration.

Passive radon monitors based on solid-state nuclear thorium detectors (SSNTDs), especially CR-39, are widely used in radon and thoron research. They can be affected by external factors, such as changes in temperature, humidity, and pressure, both before and during measurements. Evaluation of the exposed detectors involves chemical treatment, the conditions of which also affect the measurement results. These phenomena were studied by Janik et al. [7]. These authors concluded that (1) track density dependence of the age of production was statistically insignificant; and (2) simple pretreatment methods using hot water and carbon dioxide could be applied to improve the performance of nuclear track detectors.

In contrast to indoor radon concentration, which ranges from tens to thousands of Bq·m${}^{-3}$, radon activity concentration in outdoor air is in the order of a few Bq·m${}^{-3}$. Implementing large-scale radon monitoring networks to provide concentration data for environmental sciences and ensuring their comparability requires calibration techniques for radon monitors in this concentration range, which is one of the tasks of the EU traceRadon project. Mertes et al. [8] presented a novel approach to standardizing low-level radon emanation. The technique was based on integrating the radon source directly with an α-particle detector to facilitate the continuous monitoring of radon residues.

Seven articles relate to the second topic of this Special Issue, i.e., radon and thoron measurements and dose assessment.

Aghdam et al. [9] focused on the identification of radon and thoron prone areas within southeastern Ireland. They analyzed the natural radioactivity levels and radon and thoron exhalation rates of twelve geological formations and ten soil types using available indoor radon concentration data, airborne radiometric and stream sediment geochemistry, laboratory radon and thoron activity tests, and in situ dose measurements. The maps developed in this study can be used to assess radon risk in unpopulated and urban areas, as they are not dependent on the availability of indoor radon measurements. Additionally, the method can be applied to other parts of Ireland or countries where aerial, geological and soil map data are available.

Another research study which evaluated the radon hazard was presented by Giustini et al. [10]. They investigated three different areas in the central Italian region of Lazio, each of which is characterized by different radon potential levels. The authors used radon and thoron data from soil and indoor locations as well as terrestrial and indoor gamma dose rates to evaluate the effect of the individual parameters and the interplay between them to define the hazard.

The results of these two studies confirmed that the geogenic radon potential (GRP) is strictly linked to the geological setting of an area in terms of the radon source (e.g., radionuclide content of rock and soil), radon migration pathways (faults and fractures), and the mechanism of radon exhalation from soil gas to the atmosphere and indoor environment. The multivariate spatial regression models used by these two groups provide a robust solution for assessing the GRP of an area with radon in the soil as a response variable with geological and geochemical proxy variables as predictors.

Bonczyk et al. [11] presented a series of measurements of radon and thoron exhalation in the underground workings of an experimental coal mine to assess the radiation hazard to miners. Their preliminary experimental results showed a significant increase in radon levels, which may be related to the proximity of a former underground coal gasification (UCG) reactor for hydrogen production, where experiments had been conducted several years earlier. They indicated an unexpected problem related to the potential radiation hazard for those involved in experiments or regular work near the former UCG.

Wysocka et al. [12] attempted to answer the following questions: Do mine closure processes increase radon migration? How long is the period of occurrence of changes in radon concentrations in buildings after the cessation of mining activities? Results from their measurement series conducted in 2020 were compared with archival results from the 1990s. They found that in the basements of buildings where measurements were made in 1990, 2020 and 2021, the radon concentration increased significantly: the maximum values were 260 Bq·m${}^{-3}$, 644 Bq·m${}^{-3}$ and 1041 Bq·m${}^{-3}$, respectively. Analysis of the measurement results confirmed the hypothesis that local geological structures influenced the distribution of radon levels in buildings.

Two papers investigated radon and carbon dioxide measurement. In the first paper, Dovjak et al. [13] analyzed the impact of ventilation efficiency on radon and carbon dioxide concentrations in the indoor air of a small apartment located in a residential building. Based on the measurements and simulations using the CONTAM software, they determined the most appropriate design ventilation intensity (DVR) values. Their presented results will help in construction practice and legislation, leading to a safer and healthier indoor environment.

On the other hand, carbon dioxide and radon gases, which are always present in higher concentrations in underground environments than in outside air, are fundamental as tracers. Sainz et al. [14] investigated the factors driving air exchange in Altamira Cave (a World Heritage Site) over short periods. This exchange might affect the transport of materials from outside, which, in turn, could affect the conditions for preserving the cave’s rock art. They concluded that air temperature and density gradients between the indoor and outdoor atmospheres were the main driving forces behind the temporal changes in gas concentrations inside Altamira Cave. Detailed knowledge of the intensity and frequency of outgassing episodes is, on the one hand, essential as the entrance of outside air can lead to biological contamination, which is one of the most critical risks detected in caves containing art works. On the other hand, this knowledge shows that the preventive conservation of cave art is indispensable and must include a complex system involving protocols for controlling different types of risks.

Grzywa-Celińska et al. [15] studied the statistical relationship of residential radon exposure in patients with advanced lung cancer. They measured radon in dwellings in Poland of 102 patients with stage 3B or higher lung cancer. In the analyzed group, the average radon concentration during detector exposure in the patients’ living quarters was 69 Bq·m${}^{-3}$ across a range from 37 to 117 Bq·m${}^{-3}$ and it had no statistically significant effect on the type of lung cancer that developed. The lack of any statistical significance may be due to the small study group and the accompanying exposure to other harmful components, such as cigarette smoke.

Dose assessment due to ingestion intake from radon and radium in spring water was the subject of the research carried out by Yamada et al. [16]. In this study, the authors measured radon concentration in spring water in the northern part of Japan and evaluated the effective dose from water intake. The conservative estimate of the annual effective ingestion dose of 8 μ for radon and radium they obtained is much smaller than the estimated overall annual effective dose of 2.2 mSv from natural radiation in the Japanese population. However, this dosage represents 8% of the WHO individual dosing criteria of 0.1 mSv·y${}^{-1}$ for drinking water.

Despite the ever-increasing knowledge of radon and thoron behavior and, ultimately, the radiation dose to the public, scientists are still a long way from the global harmonization of radon and thoron measurements. In many countries, radon measurement procedures and action plans do not exist, and the level of radon exposure is unknown. For this reason, the broadest possible efforts must be made to promote radon measurement and to protect the general public from the radon hazard so as to improve the quality of life and mitigate the occurrence of diseases, specifically of lung cancer, which are radon related.
